# A New HPLC-MS/MS Method for the Simultaneous Determination of Quercetin and Its Derivatives in Green Coffee Beans

**DOI:** 10.3390/foods11193033

**Published:** 2022-09-30

**Authors:** Ahmed M. Mustafa, Doaa Abouelenein, Simone Angeloni, Filippo Maggi, Luciano Navarini, Gianni Sagratini, Agnese Santanatoglia, Elisabetta Torregiani, Sauro Vittori, Giovanni Caprioli

**Affiliations:** 1 School of Pharmacy, University of Camerino, Via Sant’Agostino 1, 62032 Camerino, Italy; 2 Department of Pharmacognosy, Faculty of Pharmacy, Zagazig University, Zagazig 44519, Egypt; 3 Illycaffè S.p.A., Via Flavia 110, 34147 Trieste, Italy

**Keywords:** green coffee, quercetin, flavonoids, HPLC-MS/MS, extraction methods

## Abstract

Green coffee (*Coffee arabica* and *Coffee robusta*) is one of the most commonly traded goods globally. Their beans are enriched with polyphenols and numerous health benefits are associated with their consumption. The main aim of this work was to develop a new and fast analytical HPLC-MS/MS method to simultaneously determine six flavonoid polyphenolic compounds (quercetin, rutin, isorhamnetin, quercetin-3-glucouronide, hyperoside, and quercitrin) in 22 green coffee samples from six different geographical origins (Ethiopia, Brazil, Guatemala, Nicaragua, India and Colombia). In addition, by adjusting pH, temperature, solvent type, and extraction duration, several extraction methods such as acidic and alkaline hydrolysis, and extraction without hydrolysis were evaluated. The optimal extraction procedure in terms of recovery percentages (78.67–94.09%)was acidic hydrolysis at pH 2, extraction temperature of 60 °C, extraction solvent of 70% ethanol, and extraction duration of 1.5 h. Hyperoside (878–75 μg/kg) was the most abundant compound followed by quercitrin (408–38 μg/kg), quercetin (300–36 μg/kg), rutin (238–21 μg/kg), and quercetin-3-glucouronide (225–7 μg/kg), while isorhamnetin (34–3 μg/kg) showed the lowest amount. Overall, green coffee beans are rich in flavonoid polyphenolic compounds and could be used as part of a healthy diet.

## 1. Introduction

Coffee (*Coffee arabica* and *Coffee robusta*) is one of the most popular drinks in the world [1,2]. In order to investigate the connection between coffee intake and a number of advantageous biological and medicinal qualities, research investigations on coffee ingredients are ongoing [3].

Green coffee beans are unroasted coffee beans that are higher in polyphenols and there are numerous health benefits associated with their consumption. In recent years, the functional food sector has identified polyphenols as one of the most promising ingredients because oftheir biological activity. Green coffee beans are known to have a high content of chlorogenic acid (CGA) with potential health benefits such as activity against hypertension, obesity, diabetes, etc. [4]. The most studied coffee secondary metabolites are alkaloids, such as caffeine [5]. Due to the presence of caffeine and other bioactive ingredients such as chlorogenic acids, green coffee beans were classified as a medicinal herb by the Chinese dietary system [6]. In addition, green coffee contains a variety of other phenolic components from the flavonoids’ subclass, including quercetin and its derivatives [3].

Various studies have proved the potent therapeutic potential of quercetin and its derivativesdue to their anti-inflammatory [7], antineoplastic [8], antioxidant [9,10], neuroprotective [7], antiallergic [11], and antimicrobial activities [12]. Due to these health-promoting attributes, quercetin obtained extensive approval and application in the pharmaceutical industry. Furthermore, it also has satisfactory therapeutic potential to act as anti-obesity [13], anti-diabetes [14], and to relieve Alzheimer’s disease [15,16]. Recently, when administered in conjunction with normal therapy in the early stages of viral infection, quercetin has been shown to be a safe medication that may assist to improve the early symptoms and prevent the severity of COVID-19 illness [17].

Keeping in view the health advantages, several novel extractions’ methods have been adopted for quercetin extractionin agricultural products such as ultrasound-assisted extraction [18], supercritical fluid extraction [19], microwave-assisted extraction [20], and enzymes’ extraction [15,21]. In addition, the difficulty facing the food sector is regulating food stability and quality. Therefore, it is acknowledged that techniques for analyzing quality markers of food products must be made simple and reliable. The HPLC fingerprint that uses standards is a thorough approach for evaluating the quality and reliability of food and plant extracts [22].

Generally, quantitation of quercetin ingreen coffee by HPLC/DAD or HPLC/UV–viswas not possible in many previous reports [3,10,23], except for a few studies [24,25]. Regarding HPLC-MS/MS, only one report described the quantitative determination of quercetin [26], while other researchers were unable to detect it in different green coffee varieties [5]. In addition, most of the previous studies concentrated mainly on the alkaloidal and phenolic acid contents of green coffee beans [4,24,25,27,28,29,30]. Therefore, the aims of this work were: (1) to develop a novel and quick analytical method for simultaneous determination of these flavonoid polyphenolic compounds including quercetin, rutin, isorhamnetin, quercetin-3-glucouronide, hyperoside, and quercitrin in green coffee by high-performance liquid chromatography–tandem mass spectrometry (HPLC-MS/MS) using triple quadrupole; (2) to apply this new HPLC-MS/MS method to determine all these flavonoids together for the first time in 22 green coffee samples from six different geographical origins (Ethiopia, Brazil, Guatemala, Nicaragua, India, and Colombia); (3) to assess different extraction methods such as acidic and alkaline hydrolysis, and extraction without hydrolysis, by optimizing pH, temperature, solvent type, and time of extraction, in order to choose the best method for the extraction of these flavonoids from green coffee for the first time.

## 2. Materials and Methods

### 2.1. Reagents and Standards

The analytical standards of the 6 flavonoid phenolic compounds namely, quercetin (≥95.0%, HPLC), rutin (≥95.0%, HPLC), isorhamnetin (≥95.0%, HPLC), quercetin-3-glucouronide (≥95.0%, HPLC), hyperoside (≥97.0%, HPLC), and quercitrin (≥95.0%, HPLC) were supplied by Sigma-Aldrich (Milan, Italy). Every day, suitable dilutions of the stock solutions with HPLC-grade methanol were used to create standard working solutions at various concentrations. Formic acid (99%) came from Merck (Darmstadt, Germany). Carlo Erba Reagents (Milan, Italy) provided analytical-grade hydrochloric acid (37%). Methanol suitable for HPLC was provided by Sigma-Aldrich (Milano, Italy). Deionized water was again purified (>18 MΩ cm resistivity) using a Milli-Q SP Reagent Water System (Millipore, Bedford, MA, USA). All solutions were filtered through a 0.2 μm polyamide filter obtained from Sartorius Stedim (Goettingen, Germany). All samples were filtered through Phenex™ RC 4 mm 0.2 μm syringeless filter supplied by Phenomenex (Castel Maggiore, BO, Italy), before HPLC analysis.

### 2.2. Green Coffee Samples

Illycaffè SpA located in Trieste (Italy) provided 22 wet-processed green coffee samples of *Coffea arabica* possessing different geographic origins, i.e., Ethiopia (five commercial lots: samples 1, 2, 3, 4, and 5), Brazil (five commercial lots: samples 1, 2, 3, 4, and 5), Guatemala (four commercial lots: samples 1, 2, 3, and 4), Nicaragua (four commercial lots: samples 1, 2, 3, and 4), India (two commercial lots: samples 1 and 2), and Colombia (two commercial lots: samples 1 and 2). Each sample was pulverized using a blender and kept inside a polypropylene tube (Incofar, Modena, Italy) in a dark place at room temperature.

### 2.3. Extraction Procedures and Sample Preparation 

The optimum extraction method was selected for accurately quantifying quercetin and its derivatives in various kinds of green coffee samples after several extraction techniques were evaluated. The next sections explain the three primary extraction techniques examined (acidic hydrolysis, alkaline hydrolysis, and extraction without hydrolysis), and an overview is shown in Table 1. All extractions were performed in duplicates.

#### 2.3.1. Acid Hydrolysis

Extraction of quercetins flavonoids (i.e., polyphenols) by acid hydrolysis was carried out according to Giusti, et al., 2017 [31] with some modifications. The extraction procedure began by adding 10 mL of each extraction solvent to 1 g of green coffee powder. Seven different extraction solvents (i.e., H_2_O, ethanol, ethanol 50%, ethanol 70%, methanol, methanol 50%, and methanol 70%) were tested. Then, 2 N hydrochloric acid (HCl) was added to the sample until pH adjustment to 5 or 2, and the sample was subjected to sonication for 90 min at 60 °C. Finally, different hydrolysis temperatures (25, 40, 60 °C) and extraction times (90, 180 min) were tested (Table 1). The sample was centrifuged at 5000× *g* for 10 min after the extraction, then filtered through a 0.2 µm polytetrafluoroethylene (PTFE) filter before HPLC-MS/MS injection.

#### 2.3.2. Alkaline Hydrolysis

A total of 1 g of green coffee was mixed with 10 mL of each extraction solvent (7 solvent systems were investigated) to extract the polyphenols. Then, 2 N sodium hydroxide (NaOH) was added, and the pH was adjusted to 9 and 11, after which the sample was subjected to sonication at 60 °C for 90 min. As shown in Table 1, several solvents and hydrolysis pH were tested. Prior to HPLC-MS/MS analysis, the extract was centrifuged at 5000× *g* for 10 min and filtered.

#### 2.3.3. Samples Extraction without Hydrolysis 

The extraction of 6 analytes was performed by adding 10 mL of each extraction solvent (7 solvent systems were tested) to 1 g of green coffee (Table 1). The mixture was sonicated for 90 min at 25 °C, centrifuged at 5000× *g* for 10 min and then filtered and injected into the HPLC-MS/MS. 

### 2.4. HPLC-ESI-MS/MS

Using an Agilent 1290 Infinity series and a Triple Quadrupole 6420 from Agilent Technology (Santa Clara, CA, USA) outfitted with an electrospray (ESI) source operating in negative ionization mode, quercetin and its derivatives (rutin, quercetin-3-glucouronide, hyperoside, quercetin, quercitrin, and isorhamnetin) were determined in green coffee samples using HPLC–MS/MS. Applying optimizer software (Agilent), the flow injection analysis (FIA) of the analytes (1 µL of a 1 mg L^−1^ individual standard solution) was used to optimize the HPLC-MS/MS settings. A Synergy polar-RP 80A (150 × 4.6 mmi.d., particle size 4 µm) from Phenomenex (Castel Maggiore (BO) Italy) was used to separate the polyphenols. The mobile phase was a mixture of water (A, 60%) and methanol (B, 40%), both with 0.1% formic acid, with gradient elution at a flow rate of 0.8 mL min^−1^. The solvent composition varied as follows: 0–2 min, isocratic condition, 40% B; 2–15 min, 40–80% B; 15–18 min, 80–40% B; after which, the column was reconditioned.

All solvents were filtered via a 0.2 µm filter supplied by Sartorius Stedim (Goettingen, Germany) before use, and all samples were filtered using a 0.2 µm single use syringe filter from Phenomenex (Bologna, Italy) prior to HPLC injection. Then, 5 µL was injected. The column temperature was 30 °C and the drying gas in the ionization source had a temperature of 350 °C. The capillary voltage was 4000 V, the nebulizer pressure was 55 psi, and the gas flow rate was 12 L min^−1^. Electrospray ionization (ESI)-MS in the multiple reaction monitoring (MRM) mode was used for detection. For quantification, the most prevalent product ion was utilized, and for qualification, the other product ions were utilized. Table 2 reports the chosen ion transition and the mass spectrometer parameters, including the specific time window for each molecule.

### 2.5. Statistical Analysis

Samples were analyzed in triplicates. A one-way analysis of variance (ANOVA) followed by the Tukey post hoc test were used to analyze statistical significance (*p* < 0.05). Analysis was conducted using Microsoft Excel 365 and Minitab ver. 19.0.

## 3. Results and Discussion

### 3.1. Optimization of Chromatographic Conditions

**Figure 1:**
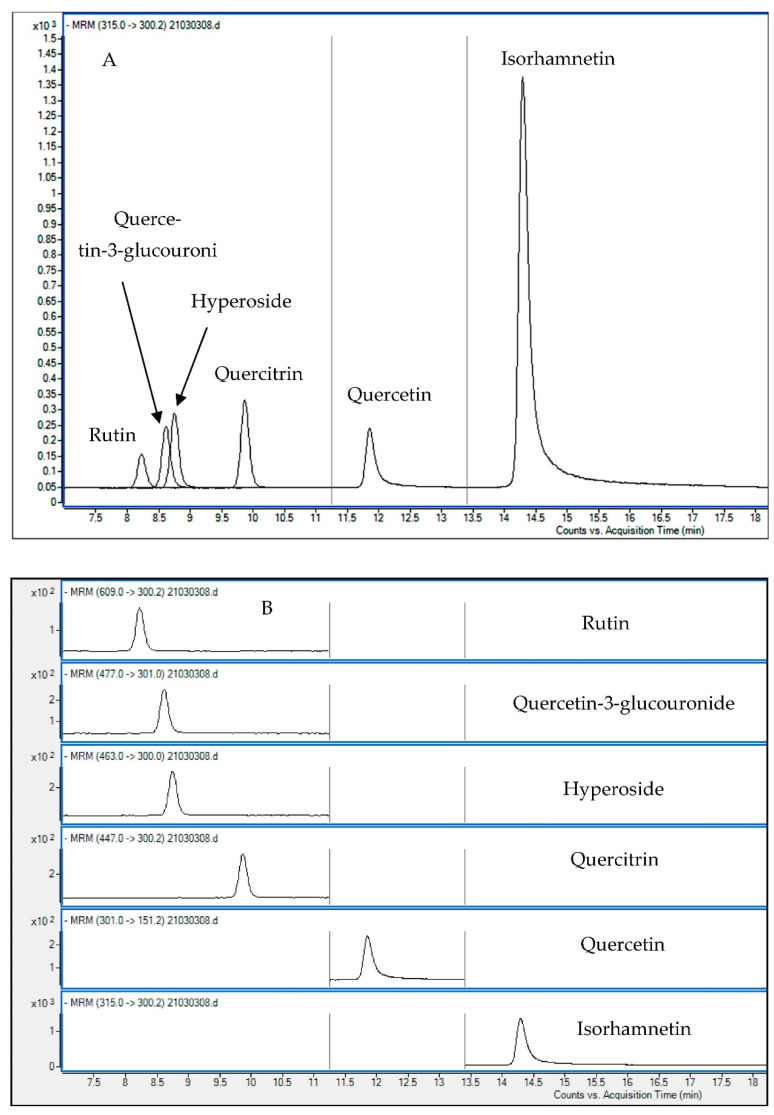
HPLC-MS/MS chromatograms of a standard mixture of the 6 analyzed quercetin derivatives (0.5 mg L^−1^) plotted as overlapped (**A**) and separate (**B**) multiple reaction monitoring (MRM) transition of each analyte.

The HPLC-MS/MS analysis in electrospray ionization (ESI) mode was carried out to ensure the accurate and unmistakable identification of the investigated compounds. The six flavonoid analytes were all analyzed concurrently in MRM mode. The MS/MS spectra and retention time were utilized to characterize analytes. The MS/MS spectra and retention times of the samples were used to describe the samples. The necessity of generatingchromatograms with the best resolution of neighboring peaks in a short time for analysis prompted the selection of the chromatographic settings. Following testing of three different columns, Synergi Polar–RP C18 (150 mm × 4.6 mm, 4 µm), Kinetex PFP (100 mm × 2.1 mm, 2.6 μm), and Zorbax (2.1 × 50 mm, 1.8 µm), Synergi Polar-RP C18 was determined to be the most effective column for the analysis and separation of this combination of molecules. Additionally, the impact of column temperatures at 25 and 30 °C was investigated. The column showed partial coelution of some compounds’ peaks at 25 °C, but when the column temperature was maintained at 30 °C, the peaks’ separation significantly improved. In order to improve the separation process in an acceptable run time, several approaches have been made. In order to do this, we used several mobile phases such as water/acetonitrile, water/methanol, both with and without formic acid to inject a standard mixture of six analytes at a concentration of 1 mg L^−1^. Water/methanol flowing at 0.8 mL min^−1^ and containing 0.1% formic acid produced the best separation. Gradient elution was also employed to produce better separation due to the diverse and vast degrees of polarity of the six standards. The baseline separation of the peaks of these six compounds were obtained with satisfactory peak symmetry under the optimal gradient circumstances utilizing gradient (0–2 min, isocratic condition, 40% B; 2–15 min, 40–80% B; 15–18 min, 80–40% B), and all compounds were eluted within 18 min. However, other gradients led to inadequate peak separation in certain cases or prolonged run times. Figure 1 displays the HPLC-MS/MS chromatogram of the standard mixture of the six analytes shown as MRM transition of each monitored component. To obtain the best MS results, ionization was completed in negative ESI mode giving precursor ions corresponding to the deprotonated [M−H]^−^ adducts. All standards’ parent-to-daughter ion transitions were tracked using the multiple reaction monitoring (MRM) mode. By experimenting with several values of fragmentor and collision energy and selecting the optimal settings that demonstrated the maximum sensitivity, precursor ions were exposed to MS/MS studies. Following the optimization of the acquisition settings, target compound quantification was carried out (Table 2). The new HPLC-MS/MS triple quadrupole method’s excellent specificity and sensitivity allowed all the analytes in the green coffee samples to be identified.

### 3.2. Method Validation

The HPLC-MS/MS method was validated after the chromatographic conditions were optimized in terms of linearity, limits of detection (LODs), limits of quantitation (LOQs), repeatability, specificity, and recovery studies (Table 3). By injecting standard mixture solutions at the nine values of 0.001, 0.005, 0.01, 0.05, 0.1, 0.5, 1, 5, and 10 mg/L, calibration curves were created, and the six analytes shown high linearity (R^2^ ≥ 0.9957) over a broad concentration range. The signal-to-noise (S/N) ratios of 3 and 10 were used as criteria, respectively, to determine the LODs and LOQs, which were then determined by injecting successive dilutions of the corresponding standard solutions. Agilent Technology’s MassHunter Software (Santa Clara, CA, USA) was used to calculate the signal-to-noise ratio (SNR). The LOQs were determined in the range of 0.001 to 0.005 mg/L, demonstrating exceptional sensitivity. The LODs ranged from 0.0002 to 0.0017 mg/L. The HPLC-MS/MS method’s intraday precision (intraday repeatability) was verified by injecting the standard mixture solution five times each day while under the ideal circumstances. Measurements were made once daily for three days in a row to determine interday precision (interday repeatability). Relative standard deviations were used to express all of the precision measurements (RSDs). With intraday and interday fluctuations, the method demonstrated extremely good precision, with RSD (%) ranging from 2.53 to 3.28 and 0.25 to 1.91%, respectively. Using HPLC-MS/MS operating in MRM mode, high specificity was reached. By monitoring retention time stability and establishing several pairings of precursor/product ions, the method’s specificity was assessed. Each analyte’s retention time stability was examined three times over the course of three days, and the RSDs% used to represent it were always less than 1.0%.

#### 3.2.1. Comparison of the Different Extraction Methods Using Recovery Studies

In order to assess the applicability and accuracy of the developed HPLC-MS/MS method, the recoveries of the six flavonoidal polyphenols were evaluated. In each extraction method, the analyzed samples were fortified in duplicate with a standard mixture of the test compounds at known concentration (0.5 ppm). Recovery % was calculated as the ratio of analyte areas in the fortified sample before extraction (pre-spike area) and in the fortified sample before injection in HPLC-MS/MS (post-spike area) obtained through instrumental analysis.

Evaluations of the pH of hydrolysis, the type of extraction solvent, the temperature, and the extraction duration resulted in the most effective method for extracting our valuable polyphenols from green coffee samples (Table 1).

##### Optimization of Type of Extraction Solvent and pH of Extraction

In plant cells, phenolic compounds can be found both free and bound, and to quantify them, it is essential to hydrolyze the insoluble-bound phenolics from the cell wall matrix. We examined the recoveries produced by the threedistinct extraction techniques we tested: acidic hydrolysis, alkaline hydrolysis, and one without hydrolysis at a different pH. The extractions were performed with seven different solvents (i.e., H_2_O, ethanol, ethanol 50%, ethanol 70%, methanol, methanol 50%, and methanol 70%) at a temperature of 60 °C for 90 min with a fortification level of 0.5 mg kg^−1^ (Table 1). The recovery data of the six analyzed flavonoids were utilized for the comparison of the different pH of hydrolysis and type of extraction solvent, and the selection of the best one from both of them. 

Using acidic hydrolysis(method A), the recoveries at pH 2 for ethanol 70% (method A.3) were the best, in the range of 78.93–94.09%, followed by methanol 70% in the range of 65.10–81.29%, while the recoveries for other solvents were lower (Table 4). The recoveries obtained at pH 5 for ethanol 70% and methanol 70% were lower than the corresponding values at pH 2 (Table 4). 

Regarding alkaline hydrolysis (method B), the obtained recoveries at pH 9 for ethanol 70% (method B.3) ranged from 0.37 to 57.14%, and at pH 11 (method B.8), the recoveries were in the range 5.26–30.58%. For the six chemicals, all of these alkaline hydrolysis techniques produced very low recoveries (0.08–60.86%), and several standards, including quercetin and isorhamnetin, were completely undetectable (Table 5).

Using method C, without hydrolysis, the recoveries for ethanol 70% solvent were in the range of 57.92–78.38% (Table 6). Although the amounts of polyphenols extracted were smaller than those obtained with method A.3 (acidic hydrolysis at pH 2), these recoveries were quite good.

These recovery analyses indicated that method A (acidic hydrolysis at pH 2 with 70% ethanol as a solvent) appeared to be particularly effective and appropriate for extracting polyphenols from green coffee samples. These results are consistent with previous discoveries in the literature, such as that polyphenols are stable under acidic settings but weak and labile under alkaline conditions, or that a low pH value of the extraction solution can prevent the oxidation of phenolics [31,32,33]. We decided to expand our investigation of acidic hydrolysis, as described in the following section, because determining the ideal pH and solvent is crucial for the extraction of polyphenols and the pH of the extraction medium depends on the nature of the phenolic compounds to be extracted and the source of food or plant material [32].

##### Optimization of Temperature and Time of Extraction

We chose to adjust the temperature and duration of the extraction after observing that acidic hydrolysis at pH 2 in 70% ethanol provided the optimal conditions for the release of insoluble-bound and free phenolic compounds. A design with three distinct temperatures (25, 40, and 60 °C) and two sonication durations was used for the experiments (1.5 h, 3 h).

It was discovered that method A.3, with acidic hydrolysis at pH 2, extraction temperature of 60 °C, and extraction duration of 1.5 h, had the ideal temperature and sonication time by comparing the recoveries of these trials at 0.5 mg kg^−1^ as well as the total extracted area, and its recoveries were in the range of 79.55–94.09% (Table 7). Meanwhile, the percentages of recoveries at 25 and 40 °C for 1.5 h were in the range of 47.73–53.90%, and 101.54–122.78%, respectively. In addition, the percentages of recoveries at 60 °C for 3 h were in the range of 49.91–103.35%. It is also noteworthy that the amount of polyphenols extracted (i.e., the extracted area) in these extractions were lower (two–eight times lower) than that obtained with method A.3 (acidic hydrolysis at pH 2 at 60 °C using ethanol 70% for 1.5 h). This is attributable to the decomposition and further degradation of flavonoids into low-molecular-weight compounds with prolonged exposure to an acidic medium at high temperatures [34,35]. For example, the acid hydrolysis for 2 h at 80 °C with 1.2 HCl efficiently produced flavonoid aglycones from glycosides, which were no longer detectable after the 2 h hydrolysis [36]. Degradation of quercetin, due to increasing reaction time, has also been reported [37]. Therefore, compared to the other examined procedures, method A.3 was shown to be superior. Therefore, method A.3 was selected as the best extraction procedure for accurately measuring the six polyphenols in the green coffee samples.

#### 3.2.2. Matrix Effect

The presence of matrix components might alter the ionization of the analytes, lowering or boosting their response, which is one of the fundamental problems with MS/MS analysis. The matrix effect was investigated using standard solution mixture at (0.5 mg kg^−1^) and matrix-matched calibration prepared by adding the standard to the extract of green coffee samples at the same concentration. Method A.3 was chosen as the extraction method. The following equation was used to compute the signal suppression/enhancement (SSE): SSE % = (postspike area − blank area/standard area) × 100; an SSE (%) of 100 implies that there is no matrix effect, while values > 100 indicate signal enhancement, and values ˂ 100 indicate signal suppression. 

It is noteworthy that the matrix effect was negligible as the SSE% ranged from 88.28 (rutin) to 102.89 (quercetin-3-glucouronide) for the six analyzed compounds. Therefore, all the tested compounds showed very low signal suppression (rutin 88.28%, hyperoside 95.11%, quercitrin 99.87%, quercetin 97.11%, isorhamnetin 98.21%) except for quercetin-3-glucouronide which displayed a negligible signal enhancement (SSE % of 102.89). The degree of signal suppression is well established in the literature to depend on the analyte’s hydrophobicity and affinity for the stationary phase. For the more hydrophobic chemicals, the impact is often less when utilizing reverse-phase (RP) stationary packings. This can explain why rutin, the highest polar analyte, displayed the highest ion suppression effect compared to the other compounds [31,38].

Among the various extraction techniques, method A.3 not only offered the best recoveries and extracted quantities of the tested polyphenols, but it also showed very little matrix effect for all the examined compounds, and thus it was chosen for the analysis of these flavonoids in our green coffee samples.

### 3.3. Application of the HPLC-MS/MS Method to Real Green Coffee Samples

Building a global database of foods with polyphenols requires the creation of special methods to assess the polyphenol or flavonoid content of food. For the food industry to assess the authenticity and quality of plant foods and their associated products, as well as for nutritionists to evaluate the polyphenol health benefits of a vegetable diet, a comprehensive database is crucial. In this study, 22 samples of green coffee from six different countries, were successfully analyzed using the newly developed HPLC-MS/MS method for quantification of the six analyzed flavonoids. All the analytes were detected and quantified in all of the samples at different concentrations (Table 8).

The most prevalent flavonols found in plant-based diets are often quercetin, myricetin, kaempferol, and isorhamnetin [39]. In the current study, hyperoside (878–75 μg kg^−1^, with an average of 289.77 μg kg^−1^) was the most abundant compound detected in the green coffee samples followed by quercitrin (408–38 μg kg^−1^, with an average of 137.91 μg kg^−1^), quercetin (300–36 μg kg^−1^, with an average of 101.64 μg kg^−1^),rutin(238–21 μg kg^−1^, with an average of 91.45 μg kg^−1^), and quercetin-3-glucouronide (225–7 μg kg^−1^, with an average of 28.82 μg kg^−1^), while isorhamnetin (34–3 μg kg^−1^, with an average of 8.36 μg kg^−1^) showed the lowest amount. The highest levels of hyperoside, quercitrin, quercetin, rutin, quercetin-3-glucouronide, and isorhamnetin were found in the Guatemala 3, Brazil 1, Guatemala 3, Ethiopia 1, Guatemala 3, and Guatemala 1 samples, respectively. Meanwhile the lowest levels were detected in Brazil 5, Nicaragua 1, Nicaragua 1, Brazil 5, Nicaragua 4, and India 1 samples, respectively. Considering the sixtargeted flavonoids, the highest total contents were found in the Guatemala 1 sample (with an average of 865.25 μg kg^−1^), followed by green coffee samples from Ethiopia (with an average of 849.40 μg kg^−1^), Colombia (with an average of 750μg kg^−1^), India (with an average of 731.00 μg kg^−1^), and Brazil (with an average of 517.40 μg kg^−1^) origins, and the lowest total content was shown in the Nicaragua samples (with an average of 303.75 μg kg^−1^). These levels exceeded those reported in the work of Ali et al., 2022 [5], who quantified hyperoside (450–20 μg kg^−1^), rutin (160–10 μg kg^−1^), and quercitrin (80–20 μg kg^−1^) in six Yemeni green coffee beans varieties, but they were not able to detect quercetin. On the other hand, our current findings on quercetin and/or rutin are less than the levels detected in some of the previous studies [24,25,26]. Overall, green coffee beans are rich in flavonoid polyphenolic compounds and could be used as part of a healthy diet.

## 4. Conclusions

A new and fast analytical HPLC-MS/MS method was developed for simultaneous determination of six flavonoid polyphenolic compounds (quercetin, rutin, isorhamnetin, quercetin-3-glucouronide, hyperoside, and quercitrin) in 22 green coffee samples from six different geographical origins (Ethiopia, Brazil, Guatemala, Nicaragua, India, and Colombia). In addition, various extraction methods, such as extraction with and without hydrolysis, as well as acidic and alkaline hydrolysis, were examined by adjusting the pH, solvent type, temperature, and extraction duration. The optimal extraction procedure in terms of recovery percentages (79.55–94.09 percent)was acidic hydrolysis at pH 2, extraction solvent of 70% ethanol, extraction temperature of 60 °C, and extraction duration of 1.5 h. Hyperoside was the most abundant compound detected in the green coffee samples followed by quercitrin, quercetin, rutin, and quercetin-3-glucouronide, while isorhamnetin showed the lowest amount. By revealing more information on the health-promoting flavonoid polyphenolic substances present in green coffee beans, the findings of this study promote them as part of a healthy diet. 

## Figures and Tables

**Table 1:** Different extraction procedures evaluated for extraction of the 6 analyzed flavonoids in green coffee.

Procedures	Extraction Type	Solvent	pH	Time	Temperature
A.1	Acidic hydrolysis	Ethanol	2	1.5 h	60 °C
A.2	Acidic hydrolysis	Ethanol 50%	2	1.5 h	60 °C
A.3 ^a^	Acidic hydrolysis	Ethanol 70%	2	1.5 h	60 °C
A.4	Acidic hydrolysis	Methanol	2	1.5 h	60 °C
A.5	Acidic hydrolysis	Methanol 50%	2	1.5 h	60 °C
A.6	Acidic hydrolysis	Methanol 70%	2	1.5 h	60 °C
A.7	Acidic hydrolysis	H_2_O	2	1.5 h	60 °C
A.8	Acidic hydrolysis	Ethanol 70%	5	1.5 h	60 °C
A.9	Acidic hydrolysis	Methanol 70%	5	1.5 h	60 °C
A.10	Acidic hydrolysis	Ethanol 70%	2	3 h	60 °C
A.11	Acidic hydrolysis	Ethanol 70%	2	1.5 h	25 °C
A.12	Acidic hydrolysis	Ethanol 70%	2	1.5 h	40 °C
B.1	Alkaline hydrolysis	Ethanol	9	1.5 h	60 °C
B.2	Alkaline hydrolysis	Ethanol 50%	9	1.5 h	60 °C
B.3	Alkaline hydrolysis	Ethanol 70%	9	1.5 h	60 °C
B.4	Alkaline hydrolysis	Methanol	9	1.5 h	60 °C
B.5	Alkaline hydrolysis	Methanol 50%	9	1.5 h	60 °C
B.6	Alkaline hydrolysis	Methanol 70%	9	1.5 h	60 °C
B.7	Alkaline hydrolysis	H_2_O	9	1.5 h	60 °C
B.8	Alkaline hydrolysis	Ethanol 70%	11	1.5 h	60 °C
B.9	Alkaline hydrolysis	Methanol 70%	11	1.5 h	60 °C
C.1	Without hydrolysis	Ethanol 70%	7	1.5 h	25 °C
C.2	Without hydrolysis	Ethanol	7	1.5 h	25 °C
C.3	Without hydrolysis	Ethanol 50%	7	1.5 h	25 °C
C.4	Without hydrolysis	Methanol	7	1.5 h	25 °C
C.5	Without hydrolysis	Methanol 50%	7	1.5 h	25 °C
C.6	Without hydrolysis	Methanol 70%	7	1.5 h	25 °C
C.7	Without hydrolysis	H_2_O	7	1.5 h	25 °C

^a^ A.3 has been evaluated as the best extraction procedure for the analysis.

**Table 2:** Parameters acquired from the analysis in HPLC/MS-MS in MRM mode used for the analysis of quercetin and its derivatives.

Compounds	Retention Time (min)	Time Window (min)	Precursor Ion (m/z)	Product Ion (m/z)	Fragmentor (V)	Collision Energy (V)	Polarity
Rutin	8.2	7–11.25	609	300.2	170	32	Negative
Quercetin-3-glucouronide	8.63	7–11.25	477	301	136	16	Negative
Hyperoside	8.81	7–11.25	463	300	170	24	Negative
Quercitrin	9.87	7–11.25	447	300.2	160	24	Negative
Quercetin	11.89	11.25–13.4	301	151.2	145	16	Negative
Isorhamnetin	14.30	13.4–end	315	300.2	145	16	Negative

**Table 3:** Method validation data of the 6 analyzed flavonoids in green coffee beans by HPLC–MS/MS.

No.	Compounds	Conc. Range (mg/L) (9 Points)	R^2 a^	LOD (mg/L) ^b^	LOQ (mg/L) ^c^	Repeatability (RSD ^d^ %, *n* = 2)	
						Intraday	Interday
1	Rutin	0.001–10	0.9991	0.0002	0.001	1.68	2.69
2	Quercetin-3-glucouronide	0.001–10	0.9995	0.0013	0.004	1.91	3.00
3	Hyperoside	0.001–10	0.9964	0.0009	0.003	1.40	2.99
4	Quercitrin	0.001–10	0.9989	0.0008	0.002	1.82	2.53
5	Quercetin	0.001–10	0.9998	0.0017	0.005	1.46	2.57
6	Isorhamnetin	0.001–10	0.9957	0.0008	0.003	0.25	3.28

^a^ R^2^ stands for the determination coefficient. ^b^ LODs refers to limit of detection and calculated as ratio of signal to noise (S/N) = 3. ^c^ LOQs refers to limit of quantification and calculated as ratio of signal to noise (S/N) = 10. ^d^ RSD stands for the relative standard deviation.

**Table 4:** Recovery percentages (*n* = 2) for acidic hydrolysis method.

	pH 2 (Temp. 60 °C, Time 1.5 h)							pH 5 (Temp. 60 °C, Time 1.5 h)	
Compounds	(A.1) EtOH	(A.2) EtOH 50%	(A.3) EtOH 70%	(A.4) MeOH	(A.5) MeOH50%	(A.6) MeOH70%	(A.7) H_2_O	(A.8) EtOH 70%	(A.9) MeOH70%
Rutin	56.83	66.22	79.55	55.77	64.60	81.29	167.42	51.44	69.02
Quercetin-3-glucouronide	55.61	60.60	78.93	48.22	58.96	73.86	161.72	61.63	51.77
Hyperoside	72.68	76.29	79.25	71.23	62.24	75.63	131.98	59.35	57.60
Quercitrin	71.37	73.14	78.67	72.4	70.29	76.43	168.92	68.37	57.31
Quercetin	78.34	56.36	86	82.93	37.02	65.10	182.49	63.58	52.52
Isorhamnetin	66.33	51.08	94.09	83.17	43.96	66.80	177.08	66.12	47.26

**Table 5:** Recovery percentages (*n* = 2) for alkaline hydrolysis method.

	pH 9 (Temp. 60 °C, Time 1.5 h)							pH 11 (Temp. 60 °C, Time 1.5 h)	
Compounds	(B.1) EtOH	(B.2) EtOH 50%	(B.3) EtOH 70%	(B.4) MeOH	(B.5) MeOH 50%	(B.6) MeOH 70%	(B.7) H_2_O	(B.8) EtOH 70%	(B.9) MeOH 70%
Rutin	0.61	7.27	1.43	0.55	22.66	8.69	83.33	15.25	75.00
Quercetin-3-glucouronide	0.85	4.54	0.36	0.31	23.95	16	104.54	5.26	119.5
Hyperoside	0.38	4.20	0.43	0.47	14.11	10.71	180	7.42	71.44
Quercitrin	0.08	18.72	16.64	2.67	38.28	18.80	135.71	30.58	51.50
Quercetin	n.d.	50	57.14	33.33	n.d.	n.d.	n.d.	n.d.	n.d.
Isorhamnetin	n.d.	n.d.	n.d.	125	n.d.	50	n.d.	n.d.	n.d.

n.d.; not detected.

**Table 6:** Recovery percentages (*n* = 2) for extraction without hydrolysis at Temp. 25 °C.

Compounds	EtOH 70% (C.1)	EtOH (C.2)	EtOH 50% (C.3)	MeOH (C.4)	MeOH 50% (C.5)	MeOH 70% (C.6)	H_2_O (C.7)
Rutin	78.38	59.17	62.13	69.32	116	94.32	73.08
Quercetin-3-glucouronide	57.92	41.02	60.21	83.43	101.43	87.71	395.66
Hyperoside	69.07	62.51	68.82	97.05	107.92	96.34	189.50
Quercitrin	75.58	68.45	66.49	89.51	121.35	101.58	330.56
Quercetin	63.98	73.06	20.22	73.76	9.41	83.69	264.71
Isorhamnetin	69.50	63.20	29.20	72.72	13.01	92.69	n.d.

n.d.; not detected.

**Table 7:** Recovery percentages (*n* = 2) at different temperatures and the time of extraction for acidic hydrolysis of EtOH 70% at pH 2 method.

Compounds	(A.3) Temp. 60 °C, 1.5 h	(A.11) Temp. 25 °C, 1.5 h	(A.12) Temp. 40 °C, 1.5 h	(A.10) Temp. 60 °C, 3 h
Rutin	79.55	51.72	115.72	103.35
Quercetin-3-glucouronide	78.93	52.81	117.58	60.83
Hyperoside	79.25	53.90	113.59	62.63
Quercitrin	78.67	50.97	122.78	50.54
Quercetin	86	47.73	101.54	50.24
Isorhamnetin	94.09	50.37	108.48	49.91

n.d.; not detected.

**Table 8:** Content (µg kg^−1^ of DW) of flavonoid quercetin and its derivatives in green coffee samples determined by HPLC-MS/MS.

n.	Sample/Compound	Rutin	Quercetin-3-glucouronide	Hyperoside	Quercitrin	Quercetin	Isorhamnetin	Total Content
1	Ethiopia 1	237.54 ± 29.0 ^a^	25.02 ± 3.2 ^b^	693.72 ± 41.1 ^ab^	285.78 ± 42.4 ^bc^	123.86 ± 4.6 ^bcd^	11.37 ± 1.8 ^bc^	1377.29 ± 15.7 ^bc^
2	Ethiopia 2	52.79 ± 8.3 ^efg^	14.78 ± 1.6 ^b^	91.82 ± 8.0 ^d^	38.03 ± 4.9 ^g^	39.11 ± 4.6 ^fg^	5.37 ± 0.6 ^ef^	241.90 ± 1.5 ^gh^
3	Ethiopia 3	131.96 ± 4.1 ^bcd^	27.29 ± 3.2 ^b^	308.32 ± 32.1 ^cd^	167.09 ± 21.2 ^def^	118.97 ± 6.9 ^bcd^	12.32 ± 0.4 ^b^	765.96 ± 58.8 ^def^
4	Ethiopia 4	140.76 ± 16.6 ^bc^	50.03 ± 6.4 ^b^	736.79 ± 17.6 ^ab^	238.53 ± 24.4 ^cd^	255.87 ± 30.0 ^a^	12.00 ± 1.8 ^b^	1434.00 ± 39.1 ^b^
5	Ethiopia 5	120.23 ± 20.7 ^bcd^	10.23 ± 1.6 ^b^	165.50 ± 3.2 ^cd^	59.92 ± 3.3 ^g^	65.19 ± 4.5 ^efg^	7.27 ± 0.7 ^def^	428.34 ± 14.0 ^efgh^
6	Brazil 1	52.79 ± 8.3 ^efg^	13.65 ± 3.2 ^b^	439.81 ± 18.6 ^bc^	407.93 ± 94.5 ^a^	47.26 ± 2.3 ^fg^	4.74 ± 0.4 ^ef^	966.17 ± 20.6 ^cd^
7	Brazil 2	58.65 ± 8.3 ^efg^	12.51 ± 1.6 ^b^	167.76 ± 28.9 ^cd^	72.60 ± 8.1 ^fg^	35.85 ± 4.5 ^g^	4.11 ± 1.3 ^ef^	351.48 ± 33.1 ^efgh^
8	Brazil 3	46.92 ± 8.1 ^fg^	22.74 ± 3.2 ^b^	230.11 ± 17.6 ^cd^	129.06 ± 22.8 ^efg^	123.86 ± 23.0 ^bcd^	4.11 ± 0.4 ^ef^	556.80 ± 22.9 ^defgh^
9	Brazil 4	102.64 ± 12.4 ^bcdef^	26.15 ± 4.8 ^b^	209.70 ± 20.8 ^cd^	112.93 ± 19.6 ^efg^	63.56 ± 2.3 ^efg^	7.27 ± 1.3 ^def^	522.25 ± 54.0 ^defgh^
10	Brazil 5	20.53 ± 4.1 ^g^	14.78 ± 1.6 ^b^	74.81 ± 6.4 ^d^	41.48 ± 3.8 ^g^	35.85 ± 9.2 ^g^	3.47 ± 0.4 ^f^	190.94 ± 24.2 ^h^
11	Guatemala 1	87.98 ± 8.3 ^cdef^	23.88 ± 4.8 ^b^	294.72 ± 32.1 ^cd^	130.21 ± 14.5 ^efg^	127.12 ± 23.0 ^bc^	34.12 ± 2.7 ^a^	698.02 ± 85.6 ^defg^
12	Guatemala 2	90.91 ± 12.4 ^cdef^	9.10 ± 0.0 ^b^	163.23 ± 12.8 ^cd^	65.68 ± 1.6 ^fg^	86.38 ± 2.3 ^cdef^	9.79 ± 0.4 ^bcd^	425.09 ± 3.1 ^efgh^
13	Guatemala 3	126.10 ± 4.1 ^bcd^	225.15 ± 99.7 ^a^	878.49 ± 19.4 ^a^	360.68 ± 21.2 ^ab^	299.87 ± 23.0 ^a^	11.69 ± 0.5 ^b^	1901.98 ± 44.8 ^a^
14	Guatemala 4	49.85 ± 4.1 ^efd^	10.23 ± 1.6 ^b^	179.10 ± 12.8 ^cd^	111.78 ± 14.7 ^efg^	78.23 ± 4.6 ^defg^	7.27 ± 0.6 ^def^	436.45 ± 17.6 ^efgh^
15	Nicaragua 1	52.79 ± 8.3 ^efg^	14.78 ± 1.6 ^b^	100.88 ± 4.8 ^d^	38.03 ± 4.9 ^g^	35.85 ± 4.6 ^g^	4.74 ± 0.4 ^ef^	247.07 ± 7.2 ^gh^
16	Nicaragua 2	49.85 ± 4.1 ^efg^	7.96 ± 1.6 ^b^	104.28 ± 3.2 ^d^	59.92 ± 3.3 ^g^	39.11 ± 4.6 ^fg^	7.58 ± 0.9 ^cde^	268.71 ± 1.1 ^gh^
17	Nicaragua 3	55.72 ± 4.2 ^efg^	17.06 ± 4.8 ^b^	121.29 ± 1.6 ^d^	123.30 ± 1.6 ^efg^	44.00 ± 2.3 ^fg^	4.42 ± 0.9 ^ef^	365.79 ± 7.1 ^efgh^
18	Nicaragua 4	82.11 ± 0.7 ^def^	6.82 ± 0.0 ^b^	95.22 ± 3.2 ^d^	108.32 ± 5.3 ^efg^	35.85 ± 7.5 ^g^	4.42 ± 0.0 ^ef^	332.75 ± 7.9 ^fgh^
19	India 1	105.57 ± 8.2 ^bcde^	23.88 ± 1.6 ^b^	310.59 ± 12.8 ^cd^	182.07 ± 29.3 ^cde^	40.74 ± 6.9 ^fg^	3.47 ± 0.4 ^f^	666.33 ± 44.7 ^defg^
20	India 2	58.65 ± 41.5 ^efg^	35.25 ± 4.7 ^b^	459.08 ± 65.1 ^bc^	139.43 ± 14.7 ^defg^	99.41 ± 16.1 ^bcde^	4.11 ± 0.4 ^ef^	795.93 ± 23.3 ^de^
21	Colombia 1	129.03 ± 0.5 ^bcd^	18.19 ± 0.1 ^b^	233.51 ± 4.5 ^cd^	62.23 ± 2.3 ^fg^	143.42 ± 12.5 ^b^	10.74 ± 0.1 ^bcd^	597.12 ± 9.5 ^defgh^
22	Colombia 2	155.43 ± 20.7 ^b^	23.88 ± 1.6 ^b^	316.25 ± 24.0 ^cd^	99.10 ± 13.0 ^efg^	296.61 ± 4.6 ^a^	12.00 ± 0.9 ^b^	903.28 ± 9.2 ^d^

Contents of analytes are expressed as µg kg^−1^ of dried weight plant material (DW). All the data are expressed as mean ± standard deviations (*n* = 3). Means that do not share letters in the same column differ significantly (*p* < 0.05) according to Tukey’s test.

## Data Availability

The data presented in this study are available on request from the corresponding author.
